# Poly[(μ_2_-1,3-di-4-pyridyl­propane)(μ_3_-1,3-phenyl­enediacetato)­cadmium]

**DOI:** 10.1107/S1600536811041432

**Published:** 2011-10-12

**Authors:** Dong Liu, Ni-Ya Li

**Affiliations:** a College of Chemistry and Materials Science, Huaibei Normal University, Huaibei 235000, Anhui, People’s Republic of China

## Abstract

In the title compound, [Cd(C_10_H_8_O_4_)(C_13_H_14_N_2_)]_*n*_, two symmetry-related Cd atoms are bridged by two carboxyl­ate O atoms into a binuclear Cd_2_ subunit around an inversion center. The Cd atom has a distorted penta­gonal–bipyramidal environment, defined by five O atoms from three different 1,3-phenylendiacetate (1,3-pda) ligands and two N atoms from two 1,3-di-4-pyridyl­propane (bpp) ligands. Each Cd_2_ subunit is linked to four different Cd_2_ subunits by four 1,3-pda ligands and four bpp ligands, forming a two-dimensional network with rhombic grids (12.50 × 12.50 Å^2^) extending along the *ab* plane.

## Related literature

For a coordination polymer with a similar structure, see: Nagaraja *et al.* (2010 ▶). For another compound synthesized from the same components as the title compound, see: Zhang *et al.* (2009 ▶). For Cd—O and Cd—N bond lengths in related structures, see: Clegg *et al.* (1995 ▶); Tao *et al.* (2000 ▶). 
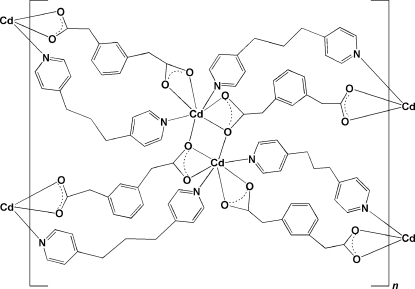


## Experimental

### 

#### Crystal data

[Cd(C_10_H_8_O_4_)(C_13_H_14_N_2_)]
*M* *_r_* = 502.84
Orthorhombic, 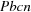

*a* = 22.573 (5) Å
*b* = 10.729 (2) Å
*c* = 17.024 (3) Å
*V* = 4123.0 (14) Å^3^
*Z* = 8
Mo *K*α radiation
μ = 1.09 mm^−1^
*T* = 223 K
0.25 × 0.25 × 0.20 mm

#### Data collection

Rigaku MercuryCCD area-detector diffractometer
Absorption correction: multi-scan (*REQAB*; Jacobson, 1998 ▶) *T*_min_ = 0.772, *T*_max_ = 0.811
13963 measured reflections
4693 independent reflections
3718 reflections with *I* > 2σ(*I*)
*R*_int_ = 0.044

#### Refinement

*R*[*F* ^2^ > 2σ(*F* ^2^)] = 0.061
*wR*(*F* ^2^) = 0.108
*S* = 1.19
4693 reflections
271 parameters
H-atom parameters constrained
Δρ_max_ = 0.65 e Å^−3^
Δρ_min_ = −0.51 e Å^−3^

### 

Data collection: *CrystalClear* (Rigaku, 2001 ▶); cell refinement: *CrystalClear*; data reduction: *CrystalStructure* (Rigaku/MSC, 2004 ▶); program(s) used to solve structure: *SHELXTL* (Sheldrick, 2008 ▶); program(s) used to refine structure: *SHELXTL*; molecular graphics: *SHELXTL*; software used to prepare material for publication: *SHELXTL* and *PLATON* (Spek, 2009 ▶).

## Supplementary Material

Crystal structure: contains datablock(s) I, global. DOI: 10.1107/S1600536811041432/aa2028sup1.cif

Structure factors: contains datablock(s) I. DOI: 10.1107/S1600536811041432/aa2028Isup2.hkl

Additional supplementary materials:  crystallographic information; 3D view; checkCIF report

## Figures and Tables

**Fig. 1.**
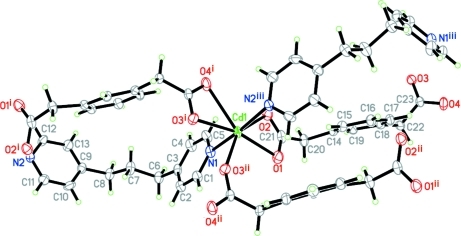
Coordination environment of Cd(II) in the title compound with nonhydrogen atoms represented by thermal ellipsoids at 30% probability level, hydrogen atoms are drawn as spheres of arbitrary radius. [Symmetry codes: (i) x + 1/2, - y + 1/2, - z; (ii) - x + 1/2, y - 1/2, z; (iii) x - 1/2, - y + 1/2, - z.]

**Fig. 2.**
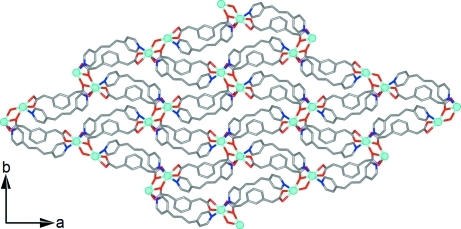
View of the two-dimensional network of the title compound extending along the ab plane.

## supplementary crystallographic information

### Comment

In view of progress of crystal engineering, the appropriate choice of metal ions and organic building blocks is the most effective and facile method to assemble metal-organic compounds with various structures (Clegg *et al.*, 1995; Nagaraja *et al.*, 2010; Tao *et al.*, 2000). In the past decade, rigid dicaboxylate and dipyridyl ligands have been widely employed as organic linkers to afford coordination polymers (Tao *et al.*, 2000). Recently, flexible dicaboxylate and dipyridyl ligands have also been used to bond with metal ions (Nagaraja *et al.*, 2010). And the complexes assembled by flexible ligands usually exhibit different structures with those of the complexes assembled by rigid ligands. In this work, we employed Cd(NO_3_)_2_ and two flexible ligands (1,3-phenylendiacetic acid, 1,3-pda, and 1,3-di-4-pyridylpropane, bpp) as our system and obtained the title compound.

As shown in Fig. 1, the symmetry-unique Cd atom is located in a pentagonal-bipyramidal environment, coordinated by five O atoms from three different 1,3-pda ligands at the equatorial sites and two N atoms from two bpp ligands at the axial sites. The Cd1–O [2.302 (3)–2.662 (3) Å] and Cd–N [2.319 (4)–2.320 (4) Å] distances are consistent with those previously observed in the related reported complexes (Clegg *et al.*, 1995; Tao *et al.*, 2000). Two symmetry related Cd atoms (Cd1 and Cd1^i^; symmetry code: (i) -*x* + 1, -*y*, -*z*) are bridged by two carboxylate O atoms into a binuclear Cd_2_ subunit around an inversion center. Each Cd_2_ subunit is linked to four different Cd_2_ subunits by four 1,3-pda ligands and four bpp ligands forming a two-dimensional (4,4) network with rhombic grids (12.50 × 12.50 Å^2^) extending along the *ab* plane (Fig. 2).

It should be noted that the complex [Cd_2_(1,3-pda)_2_(bpp)_3_]_n_ (Zhang *et al.*, 2009) was synthesized from the same components as the title compound. However, its structure is completely different.

### Experimental

Cd(NO_3_)_2_^.^4H_2_O (31 mg, 0.1 mmol), 1,3-phenylenediacetic acid (19 mg, 0.1 mmol), 1,3-di-4-pyridylpropane (20 mg, 0.1 mmol), 1.5 ml of H_2_O and 1.5 mL of EtOH were loaded to a 10 mL Pyrex glass tube. The tube was sealed and heated in an oven to 438 K for three days, and then cooled to ambient temperature at a rate of 5 K/h to form colourless blocks of the title compound, which were washed with ethanol and dried in air. Yield: 39 mg (78% yield based on Cd). Anal. calcd. for C_23_H_22_CdN_2_O_4_: C, 54.94; H, 4.41; N, 5.57. Found: C, 55.06; H, 4.22; N, 5.61. IR (KBr, cm^-1^): 1606 (*s*), 1558 (*s*), 1540 (*s*), 1418 (*m*), 1395 (*s*), 1323 (*m*), 1217 (*m*), 1108 (*m*), 1068 (*w*), 1016 (*m*), 960 (*s*), 879 (*m*), 834 (*s*), 782 (*s*), 724 (*s*), 610 (*m*), 556 (*s*).

### Refinement

All H atoms were placed in geometrically idealized positions (C–H = 0.94 Å for phenyl/pyridyl groups and C–H = 0.98 Å for methylene groups) and constrained to ride on their parent atoms with *U*_iso_(H) = 1.2*U*_eq_(C).

### Crystal data

**Table:**

[Cd(C_10_H_8_O_4_)(C_13_H_14_N_2_)]	*F*(000) = 2032
*M**_r_* = 502.84	*D*_x_ = 1.620 Mg m^−^^3^
Orthorhombic, *P**b**c**n*	Mo *K*α radiation, λ = 0.71073 Å
Hall symbol: -P 2n 2ab	Cell parameters from 9088 reflections
*a* = 22.573 (5) Å	θ = 3.1–27.5°
*b* = 10.729 (2) Å	µ = 1.09 mm^−^^1^
*c* = 17.024 (3) Å	*T* = 223 K
*V* = 4123.0 (14) Å^3^	Block, colourless
*Z* = 8	0.25 × 0.25 × 0.20 mm

### Data collection

**Table:**

Rigaku MercuryCCD area-detector diffractometer	4693 independent reflections
Radiation source: fine-focus sealed tube	3718 reflections with *I* > 2σ(*I*)
graphite	*R*_int_ = 0.044
ω scans	θ_max_ = 27.5°, θ_min_ = 3.2°
Absorption correction: multi-scan (*REQAB*; Jacobson, 1998)	*h* = −25→29
*T*_min_ = 0.772, *T*_max_ = 0.811	*k* = −13→12
13963 measured reflections	*l* = −12→22

### Refinement

**Table:**

Refinement on *F*^2^	Primary atom site location: structure-invariant direct methods
Least-squares matrix: full	Secondary atom site location: difference Fourier map
*R*[*F*^2^ > 2σ(*F*^2^)] = 0.061	Hydrogen site location: inferred from neighbouring sites
*wR*(*F*^2^) = 0.108	H-atom parameters constrained
*S* = 1.19	*w* = 1/[σ^2^(*F*_o_^2^) + (0.0287*P*)^2^ + 4.0115*P*] where *P* = (*F*_o_^2^ + 2*F*_c_^2^)/3
4693 reflections	(Δ/σ)_max_ = 0.001
271 parameters	Δρ_max_ = 0.65 e Å^−^^3^
0 restraints	Δρ_min_ = −0.51 e Å^−^^3^

### Special details

**Table:**

Geometry. All e.s.d.'s (except the e.s.d. in the dihedral angle between two l.s. planes) are estimated using the full covariance matrix. The cell e.s.d.'s are taken into account individually in the estimation of e.s.d.'s in distances, angles and torsion angles; correlations between e.s.d.'s in cell parameters are only used when they are defined by crystal symmetry. An approximate (isotropic) treatment of cell e.s.d.'s is used for estimating e.s.d.'s involving l.s. planes.
Refinement. Refinement of *F*^2^ against ALL reflections. The weighted *R*-factor *wR* and goodness of fit *S* are based on *F*^2^, conventional *R*-factors *R* are based on *F*, with *F* set to zero for negative *F*^2^. The threshold expression of *F*^2^ > σ(*F*^2^) is used only for calculating *R*-factors(gt) *etc*. and is not relevant to the choice of reflections for refinement. *R*-factors based on *F*^2^ are statistically about twice as large as those based on *F*, and *R*– factors based on ALL data will be even larger.

### Fractional atomic coordinates and isotropic or equivalent isotropic displacement parameters (Å^2^)

**Table:**

	*x*	*y*	*z*	*U*_iso_*/*U*_eq_	
Cd1	0.434747 (13)	0.10920 (3)	0.01591 (2)	0.03461 (12)	
N1	0.49203 (16)	0.2373 (3)	0.0952 (3)	0.0393 (10)	
N2	0.86922 (15)	0.4861 (3)	0.0701 (2)	0.0377 (9)	
O1	0.36029 (14)	0.1141 (3)	0.1164 (2)	0.0498 (9)	
O2	0.35892 (13)	0.2637 (3)	0.0268 (2)	0.0445 (9)	
O3	0.01992 (13)	0.4305 (3)	0.0605 (2)	0.0441 (9)	
O4	−0.02352 (13)	0.2645 (3)	0.1068 (2)	0.0525 (10)	
C1	0.5298 (2)	0.1956 (4)	0.1497 (3)	0.0451 (13)	
H1	0.5315	0.1093	0.1589	0.054*	
C2	0.5662 (2)	0.2722 (4)	0.1930 (3)	0.0436 (12)	
H2	0.5921	0.2383	0.2306	0.052*	
C3	0.56421 (18)	0.4001 (4)	0.1806 (3)	0.0401 (11)	
C4	0.5244 (2)	0.4433 (4)	0.1251 (4)	0.0511 (14)	
H4	0.5209	0.5293	0.1158	0.061*	
C5	0.4898 (2)	0.3602 (4)	0.0834 (4)	0.0509 (14)	
H5	0.4637	0.3915	0.0452	0.061*	
C6	0.60680 (19)	0.4873 (4)	0.2199 (3)	0.0451 (13)	
H6A	0.6153	0.4588	0.2734	0.054*	
H6B	0.5898	0.5712	0.2227	0.054*	
C7	0.6637 (2)	0.4890 (5)	0.1712 (4)	0.0548 (15)	
H7A	0.6791	0.4038	0.1682	0.066*	
H7B	0.6537	0.5153	0.1177	0.066*	
C8	0.71221 (19)	0.5728 (5)	0.2018 (3)	0.0478 (13)	
H8A	0.7004	0.6602	0.1959	0.057*	
H8B	0.7187	0.5563	0.2577	0.057*	
C9	0.76871 (19)	0.5497 (4)	0.1569 (3)	0.0403 (11)	
C10	0.8003 (2)	0.4414 (5)	0.1711 (3)	0.0580 (15)	
H10	0.7879	0.3871	0.2112	0.070*	
C11	0.8494 (2)	0.4124 (5)	0.1272 (4)	0.0554 (15)	
H11	0.8697	0.3378	0.1378	0.066*	
C12	0.8391 (2)	0.5898 (4)	0.0562 (3)	0.0453 (13)	
H12	0.8527	0.6436	0.0164	0.054*	
C13	0.7890 (2)	0.6227 (4)	0.0970 (4)	0.0520 (14)	
H13	0.7686	0.6959	0.0835	0.062*	
C14	0.22719 (18)	0.2311 (4)	0.1101 (3)	0.0332 (10)	
C15	0.21730 (19)	0.1230 (4)	0.0675 (3)	0.0370 (11)	
H15	0.2495	0.0778	0.0472	0.044*	
C16	0.16009 (19)	0.0817 (4)	0.0549 (3)	0.0405 (11)	
H16	0.1536	0.0089	0.0255	0.049*	
C17	0.1124 (2)	0.1464 (4)	0.0851 (3)	0.0419 (12)	
H17	0.0738	0.1167	0.0764	0.050*	
C18	0.12081 (18)	0.2547 (4)	0.1281 (3)	0.0353 (10)	
C19	0.17839 (18)	0.2964 (4)	0.1378 (3)	0.0352 (10)	
H19	0.1847	0.3721	0.1643	0.042*	
C20	0.28893 (18)	0.2752 (4)	0.1328 (3)	0.0438 (12)	
H20A	0.2946	0.2599	0.1891	0.053*	
H20B	0.2911	0.3654	0.1244	0.053*	
C21	0.33970 (19)	0.2138 (4)	0.0882 (4)	0.0428 (13)	
C22	0.06922 (18)	0.3252 (5)	0.1653 (3)	0.0408 (11)	
H22A	0.0556	0.2802	0.2121	0.049*	
H22B	0.0827	0.4080	0.1819	0.049*	
C23	0.01800 (18)	0.3392 (4)	0.1080 (3)	0.0356 (11)	

### Atomic displacement parameters (Å^2^)

**Table:**

	*U*^11^	*U*^22^	*U*^33^	*U*^12^	*U*^13^	*U*^23^
Cd1	0.02357 (18)	0.03206 (18)	0.0482 (2)	0.00165 (13)	0.00060 (14)	−0.00333 (15)
N1	0.033 (2)	0.0329 (19)	0.052 (3)	0.0006 (17)	−0.0019 (18)	−0.0084 (18)
N2	0.0289 (18)	0.039 (2)	0.045 (3)	−0.0011 (17)	0.0029 (17)	−0.0001 (18)
O1	0.0412 (18)	0.0419 (18)	0.066 (3)	0.0078 (16)	0.0093 (17)	0.0030 (17)
O2	0.0383 (18)	0.0419 (18)	0.053 (3)	0.0065 (15)	0.0058 (16)	−0.0010 (17)
O3	0.0397 (18)	0.0367 (16)	0.056 (3)	−0.0065 (14)	−0.0099 (16)	0.0124 (16)
O4	0.0305 (17)	0.0446 (18)	0.082 (3)	−0.0066 (15)	−0.0007 (17)	0.0119 (18)
C1	0.038 (3)	0.042 (3)	0.055 (4)	0.001 (2)	−0.002 (2)	−0.002 (2)
C2	0.035 (2)	0.048 (3)	0.048 (3)	0.000 (2)	−0.005 (2)	−0.005 (2)
C3	0.029 (2)	0.045 (3)	0.046 (3)	0.000 (2)	0.007 (2)	−0.013 (2)
C4	0.045 (3)	0.032 (2)	0.077 (5)	0.001 (2)	−0.007 (3)	−0.005 (3)
C5	0.039 (3)	0.045 (3)	0.069 (4)	0.002 (2)	−0.011 (3)	−0.001 (3)
C6	0.038 (3)	0.048 (3)	0.049 (4)	−0.007 (2)	0.002 (2)	−0.014 (2)
C7	0.040 (3)	0.061 (3)	0.064 (4)	−0.008 (3)	0.011 (3)	−0.021 (3)
C8	0.040 (3)	0.052 (3)	0.052 (4)	−0.006 (2)	0.008 (2)	−0.012 (2)
C9	0.036 (2)	0.042 (3)	0.042 (3)	−0.008 (2)	0.002 (2)	−0.006 (2)
C10	0.067 (3)	0.061 (3)	0.046 (4)	0.012 (3)	0.016 (3)	0.025 (3)
C11	0.051 (3)	0.057 (3)	0.059 (4)	0.013 (3)	0.009 (3)	0.017 (3)
C12	0.049 (3)	0.035 (2)	0.051 (4)	0.003 (2)	0.018 (2)	0.007 (2)
C13	0.051 (3)	0.039 (3)	0.066 (4)	0.011 (2)	0.015 (3)	0.010 (3)
C14	0.030 (2)	0.038 (2)	0.032 (3)	0.0020 (19)	0.0026 (19)	0.0021 (19)
C15	0.036 (2)	0.032 (2)	0.042 (3)	0.0040 (19)	0.003 (2)	−0.004 (2)
C16	0.040 (3)	0.036 (2)	0.045 (3)	−0.004 (2)	0.000 (2)	−0.005 (2)
C17	0.029 (2)	0.046 (3)	0.051 (4)	−0.001 (2)	−0.002 (2)	0.007 (2)
C18	0.031 (2)	0.042 (2)	0.033 (3)	0.008 (2)	0.001 (2)	0.008 (2)
C19	0.034 (2)	0.036 (2)	0.036 (3)	0.0040 (19)	−0.002 (2)	−0.0005 (19)
C20	0.030 (2)	0.046 (3)	0.056 (4)	0.002 (2)	0.005 (2)	−0.014 (2)
C21	0.028 (2)	0.040 (3)	0.061 (4)	−0.001 (2)	0.001 (2)	−0.012 (2)
C22	0.029 (2)	0.055 (3)	0.038 (3)	0.012 (2)	0.003 (2)	0.005 (2)
C23	0.026 (2)	0.038 (2)	0.043 (3)	0.007 (2)	0.002 (2)	−0.004 (2)

### Geometric parameters (Å, °)

**Table:**

Cd1—O3^i^	2.302 (3)	C7—H7B	0.9800
Cd1—N2^ii^	2.319 (4)	C8—C9	1.507 (6)
Cd1—N1	2.320 (4)	C8—H8A	0.9800
Cd1—O3^iii^	2.360 (3)	C8—H8B	0.9800
Cd1—O2	2.390 (3)	C9—C13	1.365 (7)
Cd1—O1	2.399 (3)	C9—C10	1.384 (7)
N1—C5	1.334 (6)	C10—C11	1.372 (7)
N1—C1	1.337 (6)	C10—H10	0.9400
N2—C12	1.325 (6)	C11—H11	0.9400
N2—C11	1.331 (6)	C12—C13	1.373 (7)
N2—Cd1^iii^	2.319 (4)	C12—H12	0.9400
O1—C21	1.260 (6)	C13—H13	0.9400
O2—C21	1.252 (6)	C14—C15	1.385 (6)
O3—C23	1.272 (5)	C14—C19	1.388 (6)
O3—Cd1^iv^	2.302 (3)	C14—C20	1.522 (6)
O3—Cd1^ii^	2.360 (3)	C15—C16	1.382 (6)
O4—C23	1.233 (5)	C15—H15	0.9400
C1—C2	1.376 (6)	C16—C17	1.379 (6)
C1—H1	0.9400	C16—H16	0.9400
C2—C3	1.389 (6)	C17—C18	1.387 (7)
C2—H2	0.9400	C17—H17	0.9400
C3—C4	1.383 (7)	C18—C19	1.384 (6)
C3—C6	1.499 (6)	C18—C22	1.526 (6)
C4—C5	1.381 (7)	C19—H19	0.9400
C4—H4	0.9400	C20—C21	1.524 (6)
C5—H5	0.9400	C20—H20A	0.9800
C6—C7	1.528 (7)	C20—H20B	0.9800
C6—H6A	0.9800	C22—C23	1.520 (6)
C6—H6B	0.9800	C22—H22A	0.9800
C7—C8	1.510 (6)	C22—H22B	0.9800
C7—H7A	0.9800		
			
O3^i^—Cd1—N2^ii^	97.16 (12)	C7—C8—H8B	109.7
O3^i^—Cd1—N1	93.09 (13)	H8A—C8—H8B	108.2
N2^ii^—Cd1—N1	169.74 (13)	C13—C9—C10	116.0 (4)
O3^i^—Cd1—O3^iii^	70.69 (13)	C13—C9—C8	124.7 (5)
N2^ii^—Cd1—O3^iii^	95.28 (13)	C10—C9—C8	119.0 (5)
N1—Cd1—O3^iii^	88.49 (13)	C11—C10—C9	120.8 (5)
O3^i^—Cd1—O2	150.50 (12)	C11—C10—H10	119.6
N2^ii^—Cd1—O2	84.15 (12)	C9—C10—H10	119.6
N1—Cd1—O2	86.72 (12)	N2—C11—C10	122.3 (5)
O3^iii^—Cd1—O2	138.71 (11)	N2—C11—H11	118.8
O3^i^—Cd1—O1	95.43 (11)	C10—C11—H11	118.8
N2^ii^—Cd1—O1	90.74 (13)	N2—C12—C13	123.2 (5)
N1—Cd1—O1	87.86 (13)	N2—C12—H12	118.4
O3^iii^—Cd1—O1	165.43 (12)	C13—C12—H12	118.4
O2—Cd1—O1	55.08 (12)	C9—C13—C12	120.5 (5)
C5—N1—C1	117.3 (4)	C9—C13—H13	119.7
C5—N1—Cd1	118.5 (3)	C12—C13—H13	119.7
C1—N1—Cd1	124.1 (3)	C15—C14—C19	118.2 (4)
C12—N2—C11	117.2 (4)	C15—C14—C20	122.7 (4)
C12—N2—Cd1^iii^	125.8 (3)	C19—C14—C20	118.9 (4)
C11—N2—Cd1^iii^	114.4 (3)	C16—C15—C14	120.0 (4)
C21—O1—Cd1	90.3 (3)	C16—C15—H15	120.0
C21—O2—Cd1	90.9 (3)	C14—C15—H15	120.0
C23—O3—Cd1^iv^	150.1 (3)	C17—C16—C15	120.6 (4)
C23—O3—Cd1^ii^	100.6 (3)	C17—C16—H16	119.7
Cd1^iv^—O3—Cd1^ii^	109.31 (13)	C15—C16—H16	119.7
N1—C1—C2	123.6 (4)	C16—C17—C18	120.8 (4)
N1—C1—H1	118.2	C16—C17—H17	119.6
C2—C1—H1	118.2	C18—C17—H17	119.6
C1—C2—C3	119.2 (5)	C19—C18—C17	117.5 (4)
C1—C2—H2	120.4	C19—C18—C22	120.5 (4)
C3—C2—H2	120.4	C17—C18—C22	122.0 (4)
C4—C3—C2	117.1 (4)	C18—C19—C14	122.8 (4)
C4—C3—C6	120.8 (4)	C18—C19—H19	118.6
C2—C3—C6	121.9 (5)	C14—C19—H19	118.6
C5—C4—C3	120.1 (4)	C14—C20—C21	115.3 (4)
C5—C4—H4	119.9	C14—C20—H20A	108.4
C3—C4—H4	119.9	C21—C20—H20A	108.4
N1—C5—C4	122.6 (5)	C14—C20—H20B	108.4
N1—C5—H5	118.7	C21—C20—H20B	108.4
C4—C5—H5	118.7	H20A—C20—H20B	107.5
C3—C6—C7	107.7 (4)	O2—C21—O1	123.6 (4)
C3—C6—H6A	110.2	O2—C21—C20	119.4 (4)
C7—C6—H6A	110.2	O1—C21—C20	117.0 (5)
C3—C6—H6B	110.2	O2—C21—Cd1	61.6 (2)
C7—C6—H6B	110.2	O1—C21—Cd1	62.0 (2)
H6A—C6—H6B	108.5	C20—C21—Cd1	176.5 (4)
C8—C7—C6	115.5 (4)	C23—C22—C18	111.3 (4)
C8—C7—H7A	108.4	C23—C22—H22A	109.4
C6—C7—H7A	108.4	C18—C22—H22A	109.4
C8—C7—H7B	108.4	C23—C22—H22B	109.4
C6—C7—H7B	108.4	C18—C22—H22B	109.4
H7A—C7—H7B	107.5	H22A—C22—H22B	108.0
C9—C8—C7	110.0 (4)	O4—C23—O3	121.1 (4)
C9—C8—H8A	109.7	O4—C23—C22	121.7 (4)
C7—C8—H8A	109.7	O3—C23—C22	117.3 (4)
C9—C8—H8B	109.7		

Symmetry codes: (i) −*x*+1/2, *y*−1/2, *z*; (ii) *x*−1/2, −*y*+1/2, −*z*; (iii) *x*+1/2, −*y*+1/2, −*z*; (iv) −*x*+1/2, *y*+1/2, *z*.
